# Recent advances in understanding Japanese encephalitis

**DOI:** 10.12688/f1000research.19693.1

**Published:** 2019-11-13

**Authors:** Arup Banerjee, Aarti Tripathi

**Affiliations:** 1 Laboratory of Virology, Regional Centre for Biotechnology, Faridabad, Haryana, India; 2 Translational Health Science & Technology Institute, Faridabad, Haryana, India

**Keywords:** JEV, microglia activation, neuroinflammation, exosome, blood brain barrier.

## Abstract

Japanese encephalitis (JE) is a clinical manifestation of the brain inflammation caused by JE virus (JEV). This virus imparts permanent neurological damage, thus imposing a heavy burden on public health and society. Neuro-inflammation is the hallmark of JEV infection. The prolonged pro-inflammatory response is due primarily to microglial activation, which eventually leads to severe encephalitis. A continual effort is going on in the scientific community toward an understanding of cellular and molecular factors that are involved in JEV neuro-invasion and inflammatory processes. This review not only gives a comprehensive update on the recent advances on understanding virus structure and mechanisms of pathogenesis but also briefly discusses crucial unresolved issues. We also highlight challenging areas of research that might open new avenues for controlling virus-induced neuro-inflammation.

## Changing epidemiological patterns of Japanese encephalitis virus: a significant health concern

Japanese encephalitis (JE) is a severe manifestation of inflammation in the central nervous system (CNS). JE is caused by the mosquito-borne JE virus (JEV). At present, JE is prevalent mostly in 24 countries of Southeast Asia and the Western Pacific; about 68,000 clinical cases are reported annually, and the case fatality rate is 25 to 30%. About 30 to 50% of JE survivors have permanent neurological sequelae, imposing a heavy burden on public health and society. Annual incidence of JE cases varies by age-group, with children (<15 years) being the most affected and more likely to suffer from permanent neurological sequelae than adults ^1^.

JEV follows an enzootic cycle for transmission, circulated by Culex mosquitoes ( *Culex tritaeniorhyncus* and *Culex vishnui*) and vertebrates. Vertebrates like pigs and horses act as an amplifying host, developing high titres of the virus upon infection, while the human is the dead-end host ^2^. Non-vector intra-nasal transmission as an alternative route of JEV transmission is evident in pigs and mice under experimental conditions only ^3, 4^ but still requires verification in field conditions.

Molecular genetic analysis of the JEV genome revealed five distinct clusters (G1–G5) and each cluster has a distinct distribution pattern. However, in recent years, epidemiological studies have indicated a marked change in the distribution of different JEV genotypes in endemic areas. JEV G1 has gradually replaced G3, the dominant genotype in the epidemic areas in Asia. On the other hand, G3 has spread from Asia to Europe and recently in Africa ^5^. Even JEV G2 and G5, which were reported in Malaysia, exhibited significant geographical distribution as well ^6^. Increase in travel due to trade and tourism across the globe, along with climate change, have impacted hugely on the expansion of JEV incidence in a different part of the world. Thus, the changing geographic distribution of JEV genotypes and the complications arising because of JEV infection are not only questions of concern to the scientific community but also pose public health issues.

## Viral structure: critical determinants of virulence and stability

JEV has a single-stranded positive-sense RNA genome with an enveloped capsid. The genome consists of a single 10.5-kb open reading frame (ORF) flanked by 5′ and 3′ untranslated regions (UTRs). The genome encodes a polyprotein precursor, which is co- or post-translationally cleaved by host and viral proteases into 10 different functionally active proteins: structural and non-structural (NS). Structural proteins include precursor of M (prM) (non-glycosylated), glycosylated envelope (E) protein, and capsid C protein, whereas NS proteins include NS1, NS2A, NS2B, NS3, NS4A, NS4B, and NS5. Structural proteins are required for virus entry and virion formation, whereas NS proteins are necessary for host invasion and viral replication. Recently, Wang and colleagues reported the three-dimensional structure of JEV and identified structural elements that modulate stability and virulence ^7^. The near-atomic (4.3-Å) structure of JEV clearly defines an unusual “hole” on the viral surface, and a distinct amino acid motif represents a receptor-binding site for JEV. Especially, Glu138 of E, which maps near the “holes”, is essential for binding to neuroblastoma cells. Also, the introduction of the I36F mutation on JEV membrane (M) protein confers viral attenuation and strongly impairs virus egress in mammalian cells ^8^.

Among the NS proteins, NS1 plays a role in viral replication. Structural study of the C-terminal domain of NS1 revealed extensive loop flexibility on the exterior of the protein, which may allow interactions with multiple host proteins, thus, governing host-pathogen interactions ^9^. The transmembrane domains of NS2B support both viral replication and particle formation. Several host factors that interact with viral proteins and also support viral replication have been identified. For example, TRIM52, members of tripartite motif-containing (TRIM) protein 52 and signal peptidase complex subunit 1 (SPCS1), were reported to interact with transmembrane domains of NS2A and NS2B, respectively, and influence the post-translational processing of JEV proteins and virion assembly ^10, 11^. Apart from RNA-dependent RNA polymerase (RdRp) activity, a novel function of NS5 protein in viral pathogenesis was reported. JEV NS5 interacts with mitochondrial tri-functional protein (MTP) and impairs long-chain fatty acid (LCFA) metabolism ^12^. Impaired LCFA biosynthesis can trigger proinflammatory cytokine release and contributes towards viral pathogenesis.

## Peripheral immune evasion: “camouflage” and “sabotage” mechanism

Following ingress of the virus through a mosquito bite, JEV replicates in the skin and local lymph nodes, leading to a transient low viremia in humans. During the incubation period, JEV replicates primarily in monocytes/macrophages and dendritic cells (DCs) ^13^. The infected cells then transmigrate from the periphery to the CNS, leading to inflammation in the brain ^14^. JEV must escape immune surveillance at the periphery until the necessary alterations occur at the blood–brain barrier (BBB). Several escaping mechanisms, including interference of viral antigenic peptide presentation through major histocompatibility complex (MHC) and interference in the interferon pathway, had been reported earlier ^15– 19^. A recent study suggested an enhancement in myeloid-derived suppressor cell (MDSC) populations during JEV infection ^20^. These cells inhibit T follicular helper cell–mediated immune response during JEV infection, subsequently impairing humoral immunity, which facilitates the progression of the disease in a mouse model ^20^.

DCs are antigen-presenting cells that bridge the innate and adaptive immune responses during viral infection. CD11b ^+^Ly-6C ^hi^ monocytes dampen the immune-privileged CNS significantly. A recently published study ^21^ on a mouse model suggests that CD11c ^hi^ DC promotes an imbalance in the infiltration of IL-17 ^+^Th17 to Foxp3 ^+^ regulatory T (Treg) cells and Ly-6C ^hi^ to Ly-6C ^lo^ monocytes in the CNS and promotes JE progression ^21, 22^. A contradictory role by T cells in mouse models of JE is evident. Some investigators found no role for T cells ^23, 24^ and others find a partially protective part ^23, 24^, whereas a recent report suggested complete protection against JEV ^25^. All of these reports indicate that JEV can infect and induce functional impairment of peripheral immune responses, facilitating viral survival and dissemination in the body.

## Neuro-invasion at the blood–brain barrier: Trojan horse or specific mechanism?

The CNS is segregated from peripheral tissues by the tight barrier called the BBB. Therefore, the breaching of the BBB is an essential event for CNS invasion. However, CNS viral invasions may not necessarily require initial BBB breaching. A study in the mouse model demonstrated that a Trojan horse mechanism mediated virus entry in the brain without disturbing the tight junction (TJ) proteins on the BBB. But, as the virus replicates in the brain cells, the activated microglia triggers inflammatory cytokines and chemokines that later cause BBB disruption ^26^. However, another school of thought directly supports BBB breaching as a viral entry mechanism into the CNS ^27, 28^.

A very recent study demonstrated the involvement of the mast cells (MCs) in the BBB disruption process ^29^. MCs are the hematopoietic myeloid lineage resident immune cells in the CNS and are located near the BBB and the neurovascular unit. JEV infection activates MCs, allowing the enhanced release of MC-specific protease, chymase, which in turn causes BBB leakage and facilitates virus entry into the brain ^29^.

## Mechanism of viral invasion and neuropathogenesis

In recent years, remarkable progress has been made in understanding the cellular components required for JEV entry. Apart from the CNS, the virus has been isolated from different organs of infected mice (that is, kidney, liver, and spleen), indicating that JEV also infects peripheral tissues ^30, 31^. Thus, JEV probably uses multiple cellular proteins and cell surface receptors to enter the cells. The structure of the JEV E protein contains an immunoglobulin-like E protein domain III (ED3), which has been hypothesized to interact with receptor-binding proteins ^32^. Several proteins (for example, HSP70, vimentin, laminin receptor, CD4, α5β3 integrin, and DC-SIGN) that interact with JEV E protein and play a vital role in JEV entry have been identified ^33^. Recently, a cellular protein, glucose-regulated protein 78 (GRP78), was identified as a cognate receptor for JEV in mammalian cells and plays a dual role in virus entry and replication ^34^. Another recent publication demonstrated PLVAP and GKN3 receptor proteins as critical host factors that govern JEV internalization into neurons ^35^. In the brain, the dopaminergic neuron-rich thalamus and midbrain are highly susceptible to JEV infection. It is now reported that the JEV specifically uses dopamine D2 receptor-phospholipase C to infect dopaminergic neuronal cells in human ^36^. The T-cell immunoglobulin and mucin domain 1 (TIM-1), a type I transmembrane glycoprotein, can act as an entry co-factor and significantly promotes JEV infection in non-neuronal cells ^37^.

It is interesting to note that, depending on the type of infected cells, JEV can be internalized into host cells via clathrin-dependent or clathrin-independent pathways ^38^. As compared with non-neuronal cells, neuronal cells support better viral replication. However, it is not clear whether viral entry through a clathrin-independent pathway in neuronal cells confers any advantage to establish infection in neuronal cells.

An essential aspect of JEV pathogenesis is rapid course of infection upon viral invasion in the CNS, indicating that the JEV infection develops a strategy to overcome the initial innate immune barrier. One interesting observation, as reported in ^39^, described JEV inducing activation of regulated IRE1-dependent decay pathway which selectively degrades host target transcript without affecting viral RNA, thus facilitating viral replication in mouse neuronal cells. Several host factors, including TRIM21 and activating transcription factor 3 (ATF3), have been reported to modulate cellular antiviral signalling and autophagy in JEV infected cells ^18, 19^. Autophagy is an important phenomenon induced under stressed conditions and promotes cell survival. However, in the context of JEV, the virus exploits autophagy. Autophagy was found to be functional during early stages of infection, probably induced as part of antiviral response. However, dysfunction in autophagy as indicated in blocking of autophagosome maturation was evident as infection progressed. This resulted in increased accumulation of misfolded protein ^40^, which may contribute to the development of neurological symptoms. The long term neurological disorder may develop due to the inability of JEV infected neural stem/progenitor cells to differentiate into mature neurons. It was recently reported that JEV infection evoked prolonged endoplasmic reticulum (ER) stress through ER resident chaperone GRP78, mitochondrial protein Prohibitin and heterogeneous nuclear ribonucleoprotein hnRNPC (C1/C2) and favoured neuronal stem cells apoptosis ^39, 41^, thus contributing significantly towards the development of pathogenesis.

## Microglial activation: a friend or foe?

Neuro-inflammation is the hallmark of JEV infection. JEV differentially modulates the induction of multiple pro-inflammatory mediators in human microglial cell lines ^42, 43^. Human microglia also support JEV replication. JEV can persist in microglia for up to 10 days and is capable of transmitting the virus to susceptible cells in a contact-dependent manner. Thus, infected microglia may act as a virus reservoir in the brain ^44^. Microglia play a critical role in neuronal pathologies. Depending upon stimuli, the consequence may be pro-inflammatory (M1) or anti-inflammatory (M2), resulting in neurotoxic or neuroprotective function. An early phase of microglial activation is necessary for the effective removal of infectious agents. However, prolonged microglial activation leads to the overproduction of pro-inflammatory mediators, which might override an initial beneficial effect ^45^. Recently, glutamate-mediated neurotoxicity was reported as a pathogenic event in JEV, mediated primarily by the *N*-methyl- D-aspartate receptor (NMDAR), resulting in an aberrant Ca ^2+^ influx. Excessive Ca ^2+^ flow induces mitochondrial dysfunction, leading to neuronal damage ^46^.

Apart from the release of the inflammatory milieu in response to JEV infection, change in microRNA (miRNA) profile has been reported extensively in the brain, and modulation of many of these miRNAs affects viral replication and consequent neuro-inflammation ^47– 59^.

However, until recently, it was not clear whether microglial cell–derived secreted miRNAs could influence neuronal fate. Microglial cells communicate with neighbouring cells via secreted extracellular vesicles (EVs) called exosomes ^60^. In the CNS, cross-talk between glia and neurons is crucial for a variety of biological functions ^60^. It is now established that inflammation in microglia leads to release of a distinct population of EVs. These EVs may contain several biologically active molecules (miRNAs and proteins), and transfer of those molecules to naïve neuronal cells may alter biological functions ^60, 61^. Although the role of EVs is well studied in diverse types of neurodegenerative disease, their role in JEV infection and pathogenesis remains unexplored. A recent study reported the extracellular release of *let-7a* and *let-7b* ( *let-7a/b*) via EVs from activated microglia in response to JEV infection, and transfer of such EVs to neurons, causes neuronal apoptosis via caspase activation ^62^. That study unravels a unique role of EVs in mediating pathogenesis in the brain ^62^.

## Anti-inflammatory drugs in the clinical setting: challenges and opportunities

Despite significant advances in our understanding of JE pathogenesis, the clinical development of compounds for treatment in humans is lagging. Although numerous compounds that have anti-JEV activity are available (reviewed in 63, 64), only four clinical trials against JE have been conducted in the past 10 years. Most of the compounds that were tested previously for JE in mouse models were administered either at the time of or shortly after JEV infection (that is, well before the development of symptoms) ^65, 66^. Even in a recently published study, AMG487, which inhibits receptor CXCR3, was administered in mice 12 hours before JEV infection intraperitoneally and exhibited significant improvement on the survival of JEV infected mice. As the drug was given before JEV infection, the virus was most likely cleared from the peripheral sites before it reached the brain ^67^. Also, manipulation of pro-inflammatory responses by specific drugs has also been tested. Minocycline was shown to be effective in mouse models as well as in patients, especially those who survive the initial days in hospital after onset of symptoms ^68, 69^, whereas etanercept, which blocks tumour necrosis factor alpha (TNFα) receptor, administered at days 3 and 5 after infection ^70^ could significantly improve survival of JEV-infected mice ^70^.

Apart from new drug discovery, which will take a long time to reach clinics, screening of US Food and Drug Administration (FDA)–approved drugs for antiviral activity against JEV will probably be a better option ^71^. Recently, FDA-approved drug library screening against JEV identified manidipine, as a potential JEV replication inhibitor in mouse models. Researchers also used a systems biology approach to identify potentially antiviral drugs ^72^. A proteasome inhibitor drug, bortezomib, was thus discovered, showing a significant reduction in JEV-induced lethality in mice by lessening brain damage caused by JEV infection.

Despite the advances in biological research, the CNS targeted therapy has been hindered by the reduced efficacy of molecules to cross the BBB ^73– 75^. Thus, there is an urgent need for a different approach to helping drug delivery across the BBB.

A cell-free therapeutic effect using secreted exosomes from mesenchymal stem cells (MSCs) recently showed promise against neurodegenerative diseases ^76^. Recent reports have suggested that MSC exosomes (MSC-Ex) can deliver exogenous miRNAs to neural cells and induce their differentiation, providing a solid basis for using MSC-Ex as a delivery tool to the brain ^77, 78^. MSCs can migrate to the site of injury and reduce inflammation. Also, MSCs alter BBB integrity by promoting the expression of vascular endothelial growth factor and angiogenesis ^79, 80^. In the context of JEV infection, MSC treatment itself has a beneficial effect in terms of improved recovery rate from JE in a mouse model. Interestingly, MSCs can reprogram microglia switching toward the neuroprotective state, leading to improved neuron survival ^81^.

However, the problem with the MSC-based therapy is that the stem cells often get trapped, causing obstruction of small vessels. It is now believed that the MSC-mediated effect can be mimicked by the exosomes secreted from these cells. These exosomes are very small, have low immunogenicity, and can easily reach the target site without creating the obstructive vascular effect. Thus, a cell-free therapeutic approach using MSC-derived exosomes needs to be tested against a JEV-infected animal models. Given the enormous potential of MSC-derived exosomes, more research is warranted in these areas.

## Vaccines

Given the importance of JEV burden in endemic countries, continual efforts have been made in the development of a vaccine against JEV. Currently, three different categories of licensed vaccines (mouse brain-derived inactivated, live attenuated, and genetically engineered chimeric) are available with varying efficacy (as reviewed in 82). Although SA14-14-2 live attenuated vaccine (LAV) is widely tested and observed excellent efficiency in China, Nepal, and South Korea, the effectiveness of the same vaccine was found to be lower in an Indian adult population ^83^. The reasons for the low efficacy of SA14-14-2 LAV are not clear. Several factors, including genetic background, inaccurate reporting of coverage, possible deviation from standardized assays, and absence of comparison with sera from other countries, may account for it. The latest vaccine, JENVAC, a vero cell–derived inactivated JE vaccine developed by using an Indian strain, proved durable and crossed protectivity against all commonly circulated JEV genotypes (I, II, III, and IV) ^84^. Therefore, advances in the availability and development of JEV vaccines have invigorated the scenario for JE control.

## Conclusions and future perspectives

JEV is a significant public health problem. Protection against JEV is possible through vaccination. Despite the availability of an effective vaccine against JEV, outbreaks of JE are reported in regular intervals from endemic regions. This might be due to a combination of factors, including increased reporting of JE cases or improper diagnosis, deviation of vaccines from a cold chain during storage and delivery in remote areas, and the emergence of a new strain. All of the vaccines require continuous refrigeration to maintain their potency. The thermal instability of vaccines has become a significant obstacle to vaccination programs, especially in remote areas. Thus, more research is warranted in the field of development of robust vaccine formulations with improved thermostability and immunogenicity. Given the significant change in the global risk of JE, more multi-centred, epidemiological, and post-vaccination sero-surveillance studies are desired.

In parallel, a continual search for new effective drugs is needed. It should be noted that 30 to 50% of survivors develop significant long-term neurological sequelae. Thus, a combination of drugs having antiviral and neurogenesis properties is probably a good option to treat JE.

So far, several receptors have been identified for JEV. The compounds that block entry receptors are less likely to be the right candidate for treatment since blocking of the single receptor may not be able to inhibit viral infection completely. Instead, compounds that are already approved for human use and show anti-inflammatory or anti-JE activity in animal models could be tested in pilot scale without further delay.

So far, JE research has progressed significantly in terms of the advancement of our understanding of virus structure and mechanisms of neurovirulence and pathogenesis. Further clinical and applied research is warranted for the development of more sensitive diagnostic tools and therapeutics for better managing viral encephalitis at the early stage of infection.
